# The Impact of Expressive Arts Therapy on Alexithymia Levels in Adolescent Inpatients with Severe Anorexia Nervosa

**DOI:** 10.3390/children12101394

**Published:** 2025-10-16

**Authors:** Flavia Cirillo, Giulia Spina, Mariangela Irrera, Elena Bozzola, Cristina Mascolo, Livia Gargiullo, Valentina Burla, Marco Roversi, Carla Maria Carlevaris, Stefania Dusi, Italo Pretelli, Maria Rosaria Marchili

**Affiliations:** 1 Pediatric Unit, Bambino Gesù Children’s Hospital, Istituto di Ricovero e Cura a Carattere Scientifico, 00165 Rome, Italy; flavia.cirillo@opbg.net (F.C.); giulia.spina@opbg.net (G.S.); cristina.mascolo@opbg.net (C.M.); livia.gargiullo@opbg.net (L.G.); valentina.burla@opbg.net (V.B.); mrosaria.marchili@opbg.net (M.R.M.); 2 Academy of Pediatrics, Bambino Gesù Children’s Hospital, Istituto di Ricovero e Cura a Carattere Scientifico, 00165 Rome, Italy; mariangela.irrera@opbg.net; 3 Trials Unit, Bambino Gesù Children’s Hospital, Istituto di Ricovero e Cura a Carattere Scientifico, 00165 Rome, Italy; marco.roversi@opbg.net; 4 Health Directorate, Bambino Gesù Children’s Hospital, Istituto di Ricovero e Cura a Carattere Scientifico, 00165 Rome, Italy; cmaria.carlevaris@opbg.net (C.M.C.); stefania.dusi@opbg.net (S.D.); 5 Child and Adolescent Neuropsychiatry Unit, Bambino Gesù Children’s Hospital, Istituto di Ricovero e Cura a Carattere Scientifico, 00165 Rome, Italy; italo.pretelli@opbg.net

**Keywords:** anorexia nervosa, alexithymia, theater, hospitalization, adolescent

## Abstract

Background: Anorexia nervosa (AN) is a complex psychiatric disorder that requires a multidisciplinary approach. The World Health Organization recognizes the therapeutic value of expressive arts, including drama, in enhancing emotional, cognitive, and relational domains in severe mental illnesses such as AN. Expressive arts interventions may improve emotional intelligence, empathy, and self-awareness while reducing anxiety and alexithymia. This study evaluated the impact of an adjunctive expressive arts program on alexithymia in pediatric inpatients with AN. Methods: We enrolled patients aged 11–18 years hospitalized for AN, according to the American Psychiatric Association’s Diagnostic and Statistical Manual of Mental Disorders, Fifth Edition, Text Revision, at the Pediatric Unit of Bambino Gesù Children’s Hospital, Rome. The study period ran from December 2024 to April 2025. Participants attended drama therapy sessions and expressive arts workshops in a dedicated recreational space integrated into a multidisciplinary treatment plan. Alexithymia was assessed at admission and discharge using the Toronto Alexithymia Scale (TAS-20), with scores ≥ 61 indicating alexithymia. At the end of the program, participants completed a semi-structured satisfaction questionnaire to evaluate subjective experiences and mood. Results: Thirty patients met inclusion criteria. The TAS-20 scores were statistically different between pre-/post-theater activity (*p* < 0.001). The proportion of alexithymic participants declined from 73.3% at baseline to 26.7% at discharge. Most participants reported mood improvements: 66.6% “somewhat” and 26.7% “greatly.” Additionally, 90% reported improved peer relationships. Conclusions: Expressive arts, particularly drama-based interventions, may represent an effective adjunctive therapy for adolescents with AN, supporting emotional awareness, self-regulation, and social connectedness. Arts-based interventions are associated with nonverbal avenues for emotional processing and may promote neuroplasticity, representing valuable complementary strategies for AN treatment.

## 1. Introduction

Anorexia nervosa (AN) is a severe psychiatric disorder defined by the Diagnostic and Statistical Manual of Mental Disorders, Fifth Edition (DSM-5), as a persistent restriction of energy intake, significantly low body weight, intense fear of weight gain, and body image disturbance [1]. Typically emerging in adolescence, AN is associated with high psychiatric and medical comorbidity, impairments in psychosocial functioning, and has one of the highest mortality rates among mental health disorders. Its etiology is multifactorial, involving biological, psychological, and sociocultural factors, which interact to maintain maladaptive eating behaviors and distorted body image over time [2].

The World Health Organization (WHO) recognizes the therapeutic role of expressive and theater-based arts in enhancing emotional and cognitive functioning in mental health disorders, including AN [3].

Expressive and theater-based arts can be categorized into five main types:

1. performing arts, such as music, theater, and dance;
2. visual arts, such as photography and painting;
3. literature, including creative writing and reading;
4. cultural engagement, including visits to museums and art galleries;
5. digital arts, encompassing the creation of animations and computer-generated films.

Engagement in expressive arts offers opportunities for emotional expression, regulation, and stress reduction [4]. In the context of eating and feeding disorders, these arts can serve as an effective tool both for providing motivational support and for addressing core symptoms such as distorted body image, cognitive rigidity, and low self-esteem. They have been successfully used to improve body dissatisfaction and reduce post-prandial anxiety [5]. Participating in artistic activities can also promote social interaction, which may help decrease feelings of loneliness and social isolation—factors linked to negative physiological responses, cognitive decline, loss of motor function, and mental health issues [6,7].

Mentalization refers to the capacity to understand and interpret one’s own and others’ mental states, while alexithymia is defined as the difficulty or inability to identify and describe one’s own emotions or to differentiate emotions from bodily sensations [8,9]. The literature often describes alexithymia as a deficit in affective mentalization, emphasizing the relationship between the two [10]. According to the mentalization model, AN is associated with primitive modes of thinking, in which patients struggle to differentiate between physical and emotional states, between their own experiences and those of others. They also tend to attribute causality solely to their personal experiences and interpersonal interactions [8]. These deficits may lead patients to use the somatic symptoms of AN as concrete representations of their thoughts and feelings, as well as tools for impulse and emotion regulation. Patients with AN typically exhibit lower levels of overall mentalization and higher levels of alexithymia, emotional reactivity, and emotional dysregulation [8]. In severe cases, poor emotional regulation is linked to an inability to express affective states through appropriate nonverbal cues [11].

In their review “Alexithymia and Treatment Outcome in Anorexia Nervosa”, Gramaglia, Gambaro, and Zeppegno position alexithymia as a central and relatively stable feature of AN. They argue that impaired emotional functioning and alexithymic traits are fundamental characteristics of the disorder, largely independent of depressive symptoms or illness severity, and are likely reflective of underlying emotional dysregulation [9]. While these traits may be stable, it is crucial to acknowledge that the clinical picture of severe AN is often complicated by malnutrition-induced deficits, which can exacerbate difficulties in emotional expression and engagement.

To assess alexithymia in clinical and research settings, the Toronto Alexithymia Scale (TAS-20) is one of the most widely used and validated tools. The TAS-20 is a 20-item self-report questionnaire divided into three subscales that assess difficulty in identifying emotions, difficulty in describing emotions to others, and a tendency to focus attention externally rather than on inner emotional experiences [12]. Its validity and reliability have been extensively supported. For instance, Parker confirmed the scale’s three-factor structure—Difficulty Identifying Feelings, Difficulty Describing Feelings, and Externally Oriented Thinking- using a large non-clinical sample, demonstrating its robust psychometric properties [13]. Subsequent research has broadened the application of the TAS-20 across diverse populations, uncovering meaningful associations. La Ferlita validated the scale among Italian adolescents, finding that alexithymia was associated with psychological distress and insecure attachment styles [14]. Similarly, Mori identified a connection between alexithymia and obsessive-compulsive personality traits in individuals with anxiety and depressive disorders, suggesting that it may influence treatment outcomes [15]. This growing body of research aligns with the perspective offered by Epifanio, which conceptualizes alexithymia not as a distinct symptom but as part of a broader emotional regulation disorder spanning multiple clinical conditions [16]. However, as highlighted in the 2019 WHO report on the role of the arts in health, the strength of expressive therapies lies precisely in their nonverbal and experiential modalities. These approaches can create opportunities for communication and emotional processing that bypass traditional cognitive or verbal limitations. In line with this approach, Freire reflected on the parallels between educators and artists, emphasizing the use of visual materials, such as “coded pictures”—to facilitate processes of literacy and critical awareness through art [16].

Bertolt Brecht regarded theater as a tool for critical reflection and analytical detachment [17].

Theories such as Winnicott’s concept of “play and reality” and Moreno’s work on psychodrama and spontaneity emphasize the therapeutic potential of a “potential space,” a safe, symbolic environment where patients can experiment with roles and narratives [18,19,20]. Even in the presence of cognitive impairments, such spaces may support the emergence of new coping strategies and facilitate affective integration.

Freire’s pedagogy of empowerment, Brecht’s emphasis on critical reflection through art, and Irigaray’s focus on embodied subjectivity each offer valuable frameworks for understanding how expressive therapies might function not only as therapeutic tools but also as ways to help patients regain a sense of control over their actions and participate more fully and authentically in interpersonal relationships [21].

In this context, the approach outlined by Franca Bonato in Educational Theater and Emotions offers a structured model for integrating theatrical practice into therapeutic and educational settings. Bonato’s method is organized into sequential phases designed to foster emotional awareness and interpersonal connection. It begins with a warm-up phase aimed at building trust and group cohesion through games, movement, and mirroring exercises, which help participants recognize and share emotional states in a safe environment. This is followed by a scene creation and enactment phase, in which participants explore emotions, personal experiences, and relational dynamics by performing structured or improvised scenes, alternating between the roles of actor and observer. The process concludes with a reflection and integration phase, where group discussion promotes recognition of emotional experiences, links them to real-life situations, and consolidates new coping strategies. Across all phases, the method emphasizes the use of a non-clinical, creativity-oriented space that supports playfulness, symbolic exploration, and the development of empathy and emotional intelligence [22].

Consequently, mentalization-based treatments—including expressive and theater-based arts that promote emotional awareness and reflection—may be useful for supporting patients with AN in expressing their emotions, both verbally and physically [23]. These approaches may assist patients in recognizing and understanding their own feelings, distinguishing thoughts from reality, enhancing the quality of their relationships, and developing healthier coping strategies.

Our study aimed to determine whether expressive arts reduce alexithymia in hospitalized patients with severe AN. Specifically, the primary purpose of the study was to assess whether theater and other expressive arts workshops were associated with reduced alexithymia in adolescent inpatients with AN. As a secondary aim, it sought to explore patients’ subjective experiences of the intervention through a qualitative satisfaction questionnaire, with a focus on mood, self-perception, and interpersonal relationships. The hypotheses were:

1. A structured expressive arts program reduces alexithymia levels.
2. Participants’ improvement in mood and in interpersonal relationships.
3. Nonverbal expressive channels support the development of emotional insight.

This preliminary study was conducted on a small scale to test the feasibility, methodology, and tools that could support the design of a subsequent larger and more definitive study.

## 2. Material and Methods

### 2.1. Study Design

A prospective observational study was conducted at the Pediatrics Unit of Bambino Gesù Children’s Hospital, Rome, between December 2024 and April 2025.

### 2.2. Participants

Pediatric patients aged between 11 years and under 18 years who were hospitalized for AN according to the American Psychiatric Association’s Diagnostic and Statistical Manual of Mental Disorders, Fifth Edition, Text Revision (DSM-5-TR) were eligible for inclusion [1].

The study included only patients with a diagnosis of anorexia nervosa; individuals with other eating disorders were not considered in the analysis.

For each enrolled participant, comprehensive medical, mental, and psychological data were systematically collected and recorded in a dedicated database. Specifically, for each patient, the severity of anorexia nervosa was assessed using a BMI-based index, allowing the sample to be divided into four distinct groups. Potential predisposing risk factors—such as family history of psychiatric disorders, stressful life events, and co-occurring psychiatric conditions—were also identified and documented.

Sex-disaggregated analyses were not feasible due to imbalance, and that the findings primarily apply to females.

### 2.3. Inclusion Criteria

- Age 11–18 years.
- Hospitalization ≥ 3 days.
- Confirmed AN diagnosis according to DSM-5 criteria during the pre-study screening.
- Anamnestic criteria: rapid weight loss, nutritional intake inadequate for age, refusal to drink, orthostatic presyncope, evident or reported fatigue.
- Clinical criteria: BMI ≤ 14 for patients aged 17 years and above; BMI ≤ 13.2 for patients aged 15–16 years; BMI ≤ 12.7 for patients aged 11–14 years; slowed thought processes and speech; confusion; extreme bradycardia (heart rate < 40 bpm at any time of day); tachycardia; low systolic blood pressure (<80 mmHg); blood pressure < 80/50 mmHg; orthostatic hypotension with heart rate increase >20 bpm or blood pressure decrease > 10–20 mmHg; hypothermia (<35.5 °C); hyperthermia.
- Laboratory criteria: hypoglycemia (<60 mg/dL); severe hydroelectrolytic or metabolic imbalance, in particular hypokalemia, hyponatremia, hypophosphatemia, hypomagnesemia; elevated creatinine (>100 mmol/L); cytolysis (>4× normal); leukoneutropenia (<1000/mm^3^); thrombocytopenia (<60,000/mm^3^).
- Written informed consent was provided by the patient and/or parents/legal guardians.

### 2.4. Exclusion Criteria

- Patients previously enrolled in the study.
- Physical unfitness to participate in the workshops due to clinical severity.

### 2.5. Intervention Setting and Structure

During hospitalization, patient care followed standard multidisciplinary management, involving pediatricians, child neuropsychiatrists, psychologists/psychotherapists, gastroenterologists, dietitians, and specialized nutrition nurses. Once vital signs were stabilized, patients participated in expressive arts workshops and drama therapy workshops conducted in a dedicated non-clinical space, physically separated from the inpatient ward, designed to promote creativity, playfulness, and emotional freedom. The intervention was conceptually inspired by Franca Bonato’s Educational Theater and Emotions method, which emphasizes sequential phases of warm-up, enactment, and reflection to enhance emotional intelligence and empathy [23].

The program was grounded in an integrated theoretical framework combining:

(1) Neuroscience—linking sensory input, emotional responses, and bodily representation through movement and physical expression.
(2) Psychodrama—using a “circle” format to foster non-hierarchical group dynamics, empathy, and mutual understanding.
(3) Pedagogy—applying a structured, progressive model with clear learning objectives.

Workshops were led by professionals with dual expertise in psychology and education, skilled in managing group dynamics, directing theatrical activities, and facilitating emotional and interpersonal work. Eligible participants were consecutively recruited from inpatients with severe AN (DSM-5 criteria) following neuropsychiatric assessment.

(A) Drama therapy workshop—conducted twice weekly (1 h each) and organized into two phases:

Phase 1—Warm-up: individual and collective identity: group games and mirroring exercises to foster belonging, trust, and emotional awareness; writing tasks based on primary emotions.

Phase 2—Scene enactment and reflection: performance of structured or improvised scenes exploring emotions and related behaviors, followed by group reflection to enhance emotional recognition and regulation.

(B) Expressive arts workshops—conducted five times weekly (2 h each) and organized into four phases:

Phase 1—Warm-up: sharing moods and reflections to promote trust and openness.

Phase 2—Expressive arts activity: creation using diverse media (drawing, painting, collage, sculpture, photography, storytelling, computer graphics) with a wide range of materials.

Phase 3—Sharing and reflection: presentation and discussion of artworks to encourage self-reflection and peer connection.

Phase 4—Restoring the space: tidying materials and the room as a closing ritual to promote cognitive and emotional organization.

To assess changes in alexithymia levels among patients, the Toronto Alexithymia Scale (TAS-20) was administered before the first workshop and after the conclusion of the program [18].

The scale is divided into three subscales, each of which assesses a specific aspect of alexithymia:

- DIF (Difficulty Identifying Feelings), which includes items 1, 3, 6, 7, 9, 13, 14. The subscale measures the ability to recognize one’s own emotions and distinguish them from bodily sensations.
- DDF (Difficulty Describing Feelings), including items 2, 4, 11, 12, 17 focuses on assessing the ability to communicate feelings to others.
- EOT (Externally Oriented Thinking), with items 5, 8, 10, 15, 16, 18, 19, 20, reflects a cognitive style that focuses more on external, factual events rather than introspective or emotional content.

Each item is rated on a 5-point Likert scale, where 1 = “strongly disagree” and 5 = “strongly agree.” High scores on each subscale indicate greater difficulty or tendency in the specific area.

The TAS-20 uses the following cut-off scores: a score equal to or less than 51 indicates non-alexithymia, a score equal to or greater than 61 indicates alexithymia, and scores between 52 and 60 indicate a borderline range for alexithymia [12].

Additionally, at the end of the art program, each patient completed a semi-structured qualitative satisfaction questionnaire, custom-designed by the project facilitators, to collect and analyze their subjective experiences. It included semi-open questions to assess the level of satisfaction in participating in expressive arts and theater activities and the awareness of participants’ improvement in communicating emotional states and in relationships with peers. Responses were captured on a 3-point scale: “Not at all,” “Moderately,” and “Very much.”. Additionally, the questionnaire included an open-ended section to allow participants to comment on the activities. This feedback was intended to help improve the quality of the program.

### 2.6. Ethical Procedure

Ethical approval for this study was obtained and consent for the use of medical data for research purposes by parents upon hospital admission as per institutional regulations. The study was conducted in compliance with the ethical standards outlined in the Declaration of Helsinki. In our study, we placed the highest priority on patient safety and well-being, guided by a deliberate and ethically grounded approach. The team directly involved in the project received specific training in trauma sensitivity, ensuring that all interactions with patients were conducted with care, empathy, and a commitment to maintaining a non-judgmental atmosphere. This approach ensured that the intervention was never re-traumatizing and remained fully aligned with established ethical standards.

### 2.7. Statistical Analysis

Statistical analyses were conducted using R version 4.5.1 and the statistical software R Studio version 2024.12.1.563 (Posit Team, 2025. R Studio: Integrated Development Environment for R. Posit Software, PBC, Boston, MA, USA). Categorical variables were reported as absolute frequencies and relative percentages. The TAS-20 score and all subscales were reported as mean ± standard deviation. Normality was assessed using the Shapiro–Wilk test and by visually inspecting the distributions of the variables. Comparison of TAS-20 scores before and after the intervention was performed using the paired *t*-test. Statistical significance was defined as a *p*-value ≤ 0.05. Statistical significance was defined as a *p*-value ≤ 0.05.

## 3. Results

**Figure 1:**
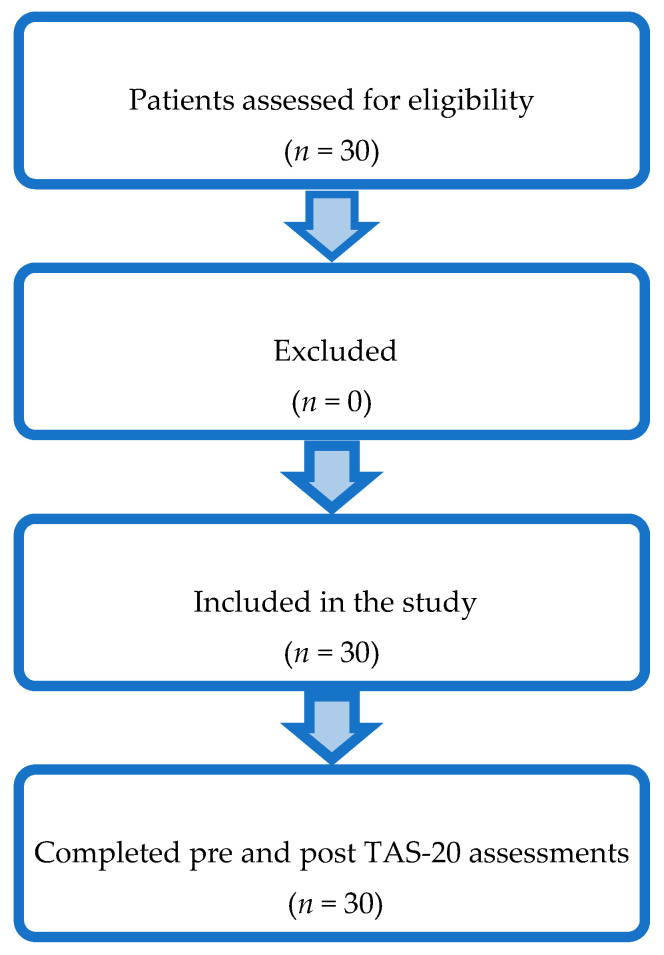
Study flow diagram illustrating the participation of patients with anorexia nervosa according to DSM-5-TR criteria.

In the study period, 30 patients (29 females; 1 male), aged 15.09 years (range: 11.96–17.36 years) and diagnosed with DSM-5-TR anorexia nervosa, fulfilled the inclusion criteria and entered the study (Figure 1). The mean BMI at admission was 14.69 ± 2.17, with 50.0% of patients classified as extreme (<15), 13.3% as severe (15–15.99), 23.3% as moderate (16–16.99), and 13.3% as mild (≥17), according to DSM-5-TR severity tiers. The mean length of stay was 19.7 ± 13.5 days (range: 6–70). As all participants completed the full three-week program, the exposure to the intervention was uniform across the sample. Each patient attended a total of six drama therapy sessions and fifteen expressive arts workshops. Moreover, all participants completed both the pre-/post-intervention TAS-20 assessments, and no missing data were recorded. Most of them (70%) were not affected by psychiatric comorbidities. Four participants reported anxiety, three dysthymia, and one disruptive behavior disorder. Notably, 7 patients (23.3%) reported life stressors, bullying (2 cases), and sexual abuse (1 case). In six cases, life stressors occurred more than 2 years before, and in 1 case during the last year. Family history was positive for anorexia nervosa (3 cases), disruptive mood dysregulation disorder (2 cases), bulimia (1 case), depressive disorder (1 case), anxiety disorder (1 case), and obsessive–compulsive disorder (1 case). Baseline clinical and demographic characteristics of the sample are summarized in Table 1, and the overall study flow is illustrated in Figure 1.

A total of 30 patients were screened for eligibility; all met inclusion criteria and were enrolled.

No dropouts or missing data occurred.

All participants completed the full intervention and both pre- and post-intervention assessments.

The mean length of hospitalization was 19.7 days, which corresponded to an exposure of five drama therapy sessions and fourteen expressive arts workshops per patient.

**Figure 2:**
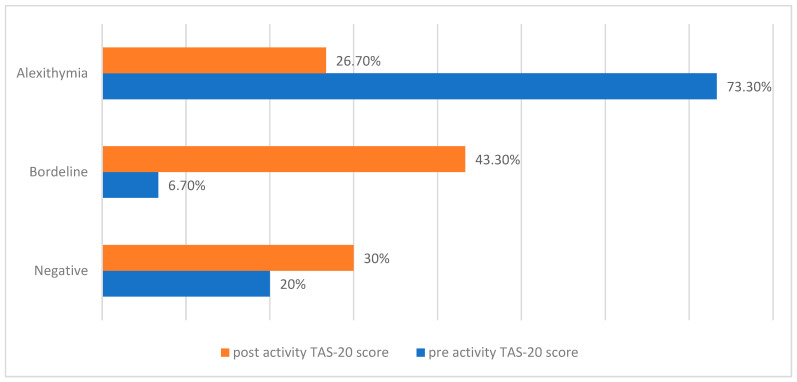
Toronto Alexithymia Scale-20 (TAS-20) total scores (mean ± SD, in points) before and after the intervention.

TAS-20 showed a statistically significant difference between pre-/post-theater activity scores (*p* < 0.001). Specifically, the pre-activity TAS-20 score was positive for alexithymia in 73.3% of participants, borderline in 6.7%, and negative in 20%. The post-activity score was indicative of alexithymia in 26.7% borderline in 43.3%, and negative in 30% of the sample (Figure 2).

Summary measures of TAS-20 and its subscale scores are presented in Table 2. Inferential details (95% CI, effect sizes) are provided in Table 2.

Pre–post differences were statistically significant (*p* = 0.004); complete estimates, including 95% confidence intervals and effect sizes (Cohen’s d), are reported in Table 2.

**Figure 3:**
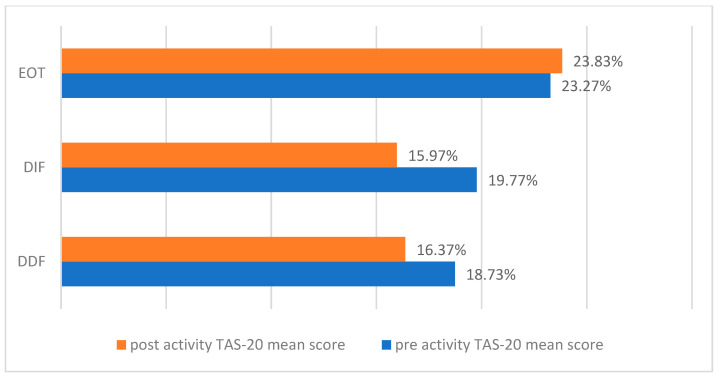
Mean (±SD) scores on the TAS-20 (Toronto Alexithymia Scale) subscales (DIF—Difficulty Identifying Feelings; DDF—Difficulty Describing Feelings; EOT—Externally Oriented Thinking)—before and after the intervention. Scores are expressed in scale points.

The Shapiro–Wilk test yielded mostly non-significant results, as confirmed by visual inspection of the statistical distributions of the variables. Therefore, paired *t*-tests were performed for all numerical variables (Figure 3).

For the DIF subscale, scores showed a slight reduction from 19.77 at baseline to 15.97 post-intervention, indicating a small improvement in the ability to recognize one’s own emotions. The DDF subscale also demonstrated a decrease from 18.73 to 16.37, reflecting a slight improvement in the ability to describe emotions to others. Conversely, the EOT subscale showed a small increase from 23.27 to 23.83, suggesting a persistent tendency toward concrete, externally oriented thinking.

**Figure 4:**
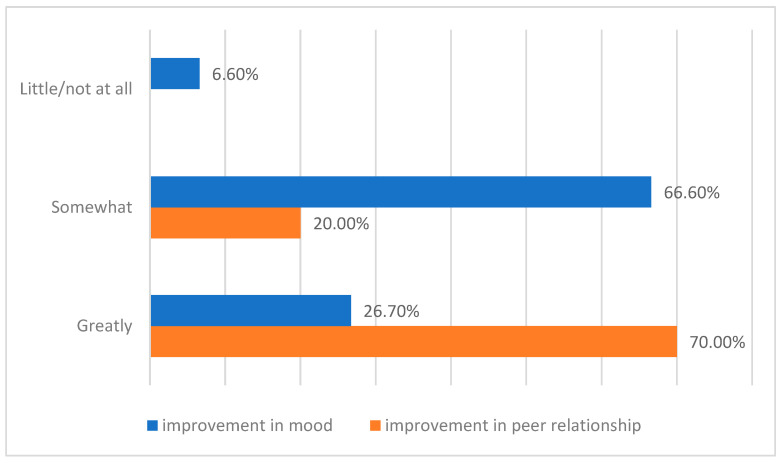
Self-reported improvement in mood and peer relationships after theater and expressive arts activities. Values are expressed as percentage (%) of participants endorsing each response category.

The satisfaction questionnaire evaluated how participants’ moods improved after engaging in theater activities as part of the therapeutic educational program. Specifically, 66.6% of participants reported that their mood had improved “somewhat,” while 26.7% said it had improved “greatly.” Only 6.6% of the sample size stated that they had received “not at all” and “a little” benefit from art workshops. Finally, nine out of ten participants confirmed an improvement in their relationships (70% “greatly,” 20% “somewhat”) (Figure 4).

Although the questionnaire included an open-ended section, the spontaneous comments provided by participants were limited and lacked sufficient detail to support a systematic thematic qualitative analysis. As a result, these responses were not included in the results.

## 4. Discussion

This research is embedded within the broader framework of therapeutic interventions that integrate expressive and performing arts—an approach endorsed by the World Health Organization for its capacity to enhance emotional and cognitive functioning in various mental health disorders, including AN [3]. Expressive arts interventions serve multiple purposes: they facilitate emotional expression, alleviate stress, and provide a creative medium to address core features of AN, such as body image distortion, cognitive rigidity, and low self-esteem.

The present study examines the impact of a structured program of theater-based and expressive arts workshops on alexithymia in adolescents hospitalized for AN, and explores potential effects on mood, emotional awareness, and interpersonal relationships. Findings suggest that such creative, non-pharmacological approaches can be a valuable adjunct to multidisciplinary treatment, contributing to reductions in alexithymia and improvements in socio-emotional functioning as reported by participants. This aligns with existing theoretical frameworks in which theater and expressive arts act as catalysts for social engagement, reducing feelings of isolation and enhancing perceived social support—factors that are critically linked to mental health outcomes [3].

In our sample, the prevalence of alexithymia, as measured by the Toronto Alexithymia Scale, decreases markedly from 73.3% at baseline to 26.7% post-intervention (*p* < 0.001), representing a reduction of nearly 50%. This finding is particularly noteworthy given that alexithymia is a well-documented and stable psychological trait in AN, often conceptualized as a maintenance factor of disorder.

Consistent with prior evidence, TAS-20 remains the most widely used self-report instrument for assessing alexithymia, with robust psychometric support [13,14,15]. In our study, both the Difficulty Identifying Feelings (DIF) and Difficulty Describing Feelings (DDF) subscales show modest reductions, reflecting a slight improvement in the ability to recognize and articulate emotions—skills considered central to AN treatment. Conversely, the Externally Oriented Thinking (EOT) subscale demonstrates a minimal increase, suggesting a transient greater focus on concrete aspects over internal emotional experiences.

Interpretation of the total TAS-20 score must consider the structure and psychometric characteristics of the instrument. The total score derives from three subscales—DIF (7 items), DDF (5 items), and EOT (8 items)—and even small shifts across subscales can cumulatively result in a more pronounced change in the total. Due to the unequal number of items, variations in subscales with more items, such as EOT, can have disproportionate influence on the total score. Moreover, group-level means can mask heterogeneity in individual responses: some participants may show substantial improvement in DIF and DDF, while others exhibit stability or less pronounced changes in EOT.

Importantly, previous research demonstrated that EOT tends to be less sensitive to short-term therapeutic effects than DIF and DDF, and presents lower internal consistency, in part due to the high proportion of reverse-scored items [24,25,26]. This psychometric limitation should be considered when interpreting EOT changes in the context of clinical trials. In this light, the slight post-intervention increase observed in EOT—despite improvements in DIF and DDF—may reflect its lower responsiveness and measurement reliability rather than a genuine deterioration in cognitive style [13,14,15].

Self-reported data are consistent with the TAS-20 findings: 93.3% of participants reported improved mood and 90% noted better relationships with peers. In inpatient settings, where social connection and group cohesion are crucial, theater may reduce isolation, build trust, and enhance emotional regulation [27,28,29,30,31].

In our cohort, 29 out of 30 participants were female and only one was male. This pronounced imbalance mirrors epidemiological findings, which estimate a female-to-male ratio in AN of approximately 11:1 [32]. This extreme disparity prevents meaningful sex- or gender-based comparisons. Such a limitation is especially pertinent within the framework of gender medicine, which emphasizes how biological sex and sociocultural gender factors shape the etiology, clinical presentation, and treatment outcomes in psychiatric disorders, including AN [33]. Future studies with larger and more balanced samples are necessary to elucidate these dynamics and inform gender-sensitive therapeutic strategies.

Finally, the relatively low rates of self-reported psychiatric comorbidities (30%) and stressful life events (23.3%) may be influenced by alexithymia-related underreporting: individuals with impaired emotional awareness may also under-detect or under-report anxiety, depression, or external stressors [16,34]. Further research is needed to determine whether extended expressive arts programs may further reduce alexithymia and enhance recognition of psychological distress.

## 5. Strengths and Limitations

The main strength of this study is the integration of theater-based expressive arts within a multidisciplinary inpatient program for adolescents with severe AN hospitalized during an acute phase of the disorder. While the Toronto Alexithymia Scale has been previously used in research on AN, the originality here lies in combining this validated tool with a structured drama therapy-based and artistic–expressive intervention in this clinical population. The program was specifically designed to address emotional dysregulation, alexithymia, and social withdrawal—core psychological features of AN—through experiential, nonverbal modalities aimed at fostering emotional recognition, regulation, and interpersonal connection. Another strength is the mixed-methods approach: combining quantitative TAS-20 results with qualitative patient-reported outcomes provides a broader understanding of the intervention’s impact. The inpatient context, with its stable and controlled environment, also favored high adherence and participation.

However, several limitations must be considered. First, the near-total predominance of females in the sample (96.7%), reflecting the well-established higher prevalence of AN in females but prevented sex- or gender-based analyses. This imbalance limits the ability to explore potential differences in presentation or treatment response across genders, underscoring the need for future studies with more balanced cohorts. The highly unbalanced sex distribution of the sample (29 females and 1 male), which did not allow analyses by sex. Therefore, the findings primarily generalize to female inpatients with anorexia nervosa. The single male case was included in the analyses, and its presence did not change the direction or magnitude of the observed effects.

Secondly, concomitant treatments—including psychopharmacological therapy, nutritional rehabilitation, and psychological support—may have influenced the outcomes, limiting attribution of effects solely to the expressive arts intervention.

Third, the follow-up period was short, as the second TAS-20 was collected just before hospital discharge, providing no information on the long-term sustainability of the observed improvements. Fourth, the use of a self-report tool may not fully capture actual changes in emotional awareness, and the lack of a structured qualitative analysis of open-ended questionnaire responses further limit interpretability. Finally, the satisfaction questionnaire is subject to potential response biases, including social desirability.

## 6. Conclusions

This study offers promising preliminary indications that structured theater activities and expressive arts may be a feasible and acceptable adjunct to inpatient treatment for adolescents with severe DSM-5-TR anorexia nervosa during the acute phase of illness. The findings point to potential signals of benefit in relation to alexithymia, with measurable movement in domains linked to emotional recognition and verbalization-the core targets of the intervention. Early patterns also suggest possible improvements in mood and interpersonal functioning, warranting further exploration. These results should be viewed primarily as feasibility and signal-detection findings, highlighting areas of promise rather than establishing causal efficacy. Larger, methodologically rigorous studies—ideally incorporating randomized controlled designs—are needed to determine whether these preliminary signals can be replicated and distinguished from non-specific therapeutic factors. Long-term follow-up would also help clarify the stability and clinical relevance of these observed trends. Future research may benefit from exploring potential mechanisms of action, including whether specific program components (e.g., improvisation, role-play, or nonverbal expression) drive effects. In summary, incorporating theater and expressive arts into multidisciplinary care for adolescents with AN appears both feasible and acceptable, providing a basis for further systematic investigation into their potential therapeutic value.

## Figures and Tables

**Table 1:** Baseline clinical and demographic characteristics of patients with anorexia nervosa.

Characteristic	Value
Age (years), mean ± SD (range)	15.09 ± SD (range 11.96–17.36)
Sex: *n* (%)	Female: 29 (96.7%) Male: 1 (3.3%)
BMI (kg/m^2^)	14.69 ± 2.17
BMI severity tiers	Mild (≥17): 4 (13.3%) Moderate (16–16.99): 7 (23.3%) Severe (15–15.99): 4 (13.3%) Extreme (<15): 15 (50.0%)
Length of stay (LOS) (days), mean ± SD (range)	19.7 ± 13.5 (range 6–70)
Psychiatric comorbidities: *n* (%)	None: 22 (73.3%)Any: 8 (26.7%) - Anxiety: 4 (13.3%) - Dysthymia: 3 (10%) - Disruptive behavior disorder: 1 (3.3%)
Life stressors	Yes: 7 (23.3%) - Parental conflict/separation: 4 (13.3%) - Bullying: 2 (6.7%) - Sexual abuse: 1 (3.3%) No: 23 (76.7%)
Life stressors	>2 years before: 6 Last year: 1
Family psychiatric history	None: 21 (70%) Any: 9 (30%) - Anorexia nervosa: 3 (10%) - Disruptive mood dysregulation disorder: 2 (6.7%) - Bulimia: 1 (3.3%) - Depression: 1 (3.3%) - Anxiety: 1 (3.3%) - Obsessive–compulsive disorder: 1 (3.3%)

Data are presented as mean ± standard deviation (SD) and range for continuous variables, and as number (percentage) for categorical variables. BMI severity tiers are defined according to DSM-5-TR criteria (Mild ≥ 17.0; Moderate 16.0–16.99; Severe 15.0–15.99; Extreme < 15.0).

**Table 2:** Summary measures of Toronto Alexithymia Scale-20 (TAS-20) and subscales. Values are presented as mean ± standard deviation (SD). Differences are expressed with 95% confidence intervals (C.I.). Difference = Pre − Post; negative values indicate improvement. C.I. = confidence interval; EOT = externally oriented thinking; DIF = difficulty identifying feelings; DDF = difficulty describing feelings.

TAS-20	Pre-Intervention (Mean ± SD)	Post-Intervention (Mean ± SD)	Difference (95% C.I.)	*p*-Value	Cohen’s d
TOTAL	63.03 ± 10.37	55.77 ± 9.31	−7.27 (−9.73, −4.81)	<0.001	−0.73
EOT	23.27 ± 3.71	23.83 ± 3.50	−0.63 (−2.54, 0.27)	<0.001	−0.31
DIF	19.77 ± 6.42	15.97 ± 6.48	−3.80 (−5.27, −2.33)	<0.001	−0.59
DDF	18.73 ± 4.38	16.37 ± 4.78	−2.37 (−3.44, −1.29)	0.109	−0.51

## Data Availability

Data supporting reported results are available upon reasonable request by contacting the corresponding authors.

## Abbreviations

AN	Anorexia Nervosa
BMI	Body Mass Index
DDF	Difficulty Describing Feelings
DIF	Difficulty Identifying Feelings
DSM-5-TR	Diagnostic and Statistical Manual of Mental Disorders, Fifth Edition, Text Revision
EOT	Externally Oriented Thinking
TAS-20	Toronto Alexithymia Scale
WHO	World Health Organization
